# Object-based selection modulates top-down attentional shifts

**DOI:** 10.3389/fnhum.2014.00090

**Published:** 2014-02-21

**Authors:** Satoshi Nishida, Tomohiro Shibata, Kazushi Ikeda

**Affiliations:** ^1^Department of Integrative Brain Science, Graduate School of Medicine, Kyoto UniversityKyoto, Japan; ^2^Graduate School of Information Science, Nara Institute of Science and TechnologyIkoma, Japan; ^3^Graduate School of Life Science and Systems Engineering, Kyushu Institute of TechnologyKitakyushu, Japan

**Keywords:** visual attention, object-based attention, attentional prioritization, attentional spreading, object-specific advantage, visual search, parallel and serial search, psychophysics

## Abstract

A large body of evidence supports that visual attention – the cognitive process of selectively concentrating on a salient or task-relevant subset of visual information – often works on object-based representation. Recent studies have postulated two possible accounts for the object-specific attentional advantage: attentional spreading and attentional prioritization, each of which modulates a bottom-up signal for sensory processing and a top-down signal for attentional allocation, respectively. It is still unclear which account can explain the object-specific attentional advantage. To address this issue, we examined the influence of object-specific advantage on two types of visual search: parallel search, invoked when a bottom-up signal is fully available at a target location, and serial search, invoked when a bottom-up signal is not enough to guide target selection and a top-down control for shifting of focused attention is required. Our results revealed that the object-specific advantage is given to the serial search but not to the parallel search, suggesting that object-based attention facilitates stimulus processing by affecting the priority of attentional shifts rather than by enhancing sensory signals. Thus, our findings support the notion that the object-specific attentional advantage can be explained by attentional prioritization but not attentional spreading.

## INTRODUCTION

A large body of evidence supports that visual attention – the cognitive process of selectively concentrating on a salient or task-relevant subset of visual information – often works on object-based representation (see Olson, 2001; Scholl, 2001; Yantis and Serences, 2003; Mozer and Vecera, 2005 for reviews). Psychophysical studies using the two-rectangle method introduced by Egly et al. (1994) have provided clear evidence for object-based attention (Egly et al., 1994; Moore et al., 1998; Avrahami, 1999; Shomstein and Yantis, 2002, 2004; Müller and Kleinschmidt, 2003; Shomstein and Behrmann, 2006; Chen and Cave, 2008; Richard et al., 2008). The studies demonstrated that, when spatial attention is directed to a location within an object (referred to as an “attended object”), the other locations within the attended object obtain object-specific attentional advantage over the other objects and locations in a scene.

Recent studies have postulated two possible accounts for the object-specific attentional advantage (Shomstein and Yantis, 2002). One account is *attentional spreading*, according to which spatial attention automatically spreads throughout an attended object (Chen and Cave, 2008; Richard et al., 2008). Hence, object boundaries determine the region of attentional spreading, which facilitates bottom-up sensory processes for visual stimuli presented within the attended object relative to stimuli presented at other locations. The other account is *attentional prioritization*, according to which visual stimuli at different locations in the visual field are attentionally prioritized depending on whether or not the stimuli are inside an attended object: stimuli within the attended object are automatically assigned higher attentional priority (without the spreading of spatial attention) than those within unattended objects or at unattended locations (Avrahami, 1999; Shomstein and Yantis, 2002, 2004). Hence, object boundaries constrain the allocation of attentional priority, which determines the order of spatially attentional shifts that are top-down covert orienting of an attentional focus toward particular stimuli or locations. Thus, the accounts of attentional spreading and attentional prioritization assume the effect of object representation on different levels of attentional processes: bottom-up sensory enhancement and top-down attentional shifts, respectively.

It is still unclear which account can explain the object-specific attentional advantage because the evidence reported so far supports both (Shomstein and Yantis, 2002, 2004; Chen and Cave, 2008; Richard et al., 2008). To further clarify this, we examined the influence of the object-specific advantage on two types of visual search: parallel search and serial search (Treisman and Gelade, 1980; Duncan and Humphreys, 1989; Wolfe et al., 1989). In the literature on visual search, the parallel search is regarded as a bottom-up attentional selection process to select salient stimuli (e.g., a red stimulus surrounded by green stimuli); the selection process is referred to as “pop-out.” Alternatively, the serial search is regarded as an attentional selection process when a bottom-up signal occurs but is not enough to guide the selection, and requires shifts of focused attention by a top-down control. We conducted a psychophysical experiment consisting of two visual search tasks that required either parallel or serial search. In the parallel and serial search tasks, search targets and distractors were presented within two segregated objects. If the attentional spreading account were correct, reaction times (RTs) would be shorter for targets located within the attended object than for targets within the unattended object (i.e., the object-specific attentional advantage) in both of the parallel and serial search tasks since attentional spreading throughout the attended object enhances the bottom-up sensory signal of a target which subsequently appears within the object (Kastner et al., 1998; Reynolds et al., 1999); even for a pop-out target in parallel search, the enhancement of the sensory signal induced by a pop-out target can strengthen the pop-out effect and facilitate the process of bottom-up attentional selection. If the attentional prioritization account were correct, the object-specific attentional advantage would be observed only in the serial search task since attentional prioritization modulates the shifts of focused attention during serial search but not the pop-out effect during parallel search. In the present study, we tested these two alternative predictions by assessing behavioral data during the parallel and serial search tasks.

## MATERIALS AND METHODS

### BEHAVIORAL TASKS

**FIGURE 1:**
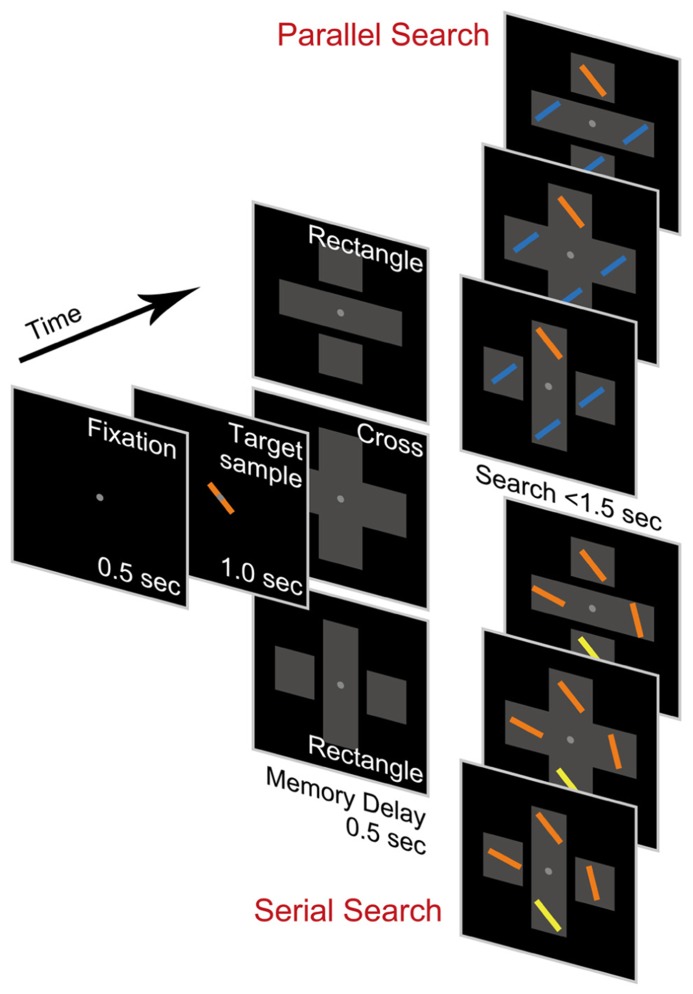
** Visual stimuli and behavioral tasks**. Tasks consisted of two types: parallel search task (upper) and serial search task (lower). In both task types, observers searched for a target among a stimulus array including four line-shaped stimuli after appearance of the target sample. The target stimulus was distinguished from distractors by color and orientation in the parallel search task but by either color or orientation in the serial search task. In the course of each task trial, task-irrelevant closure objects appeared separately (rectangle condition) or connectedly (cross condition) in the background. For illustration purposes, the stimulus colors were modified from the actual colors used in our experiments.

We conducted two different types of visual search tasks: a parallel search task and a serial search task (**Figure 1**). In each trial, participants were required to search for a target among four line-shaped stimuli (one target and three distractors) presented after the appearance of the target sample. In the parallel search task, the target had an orthogonal orientation and color opposite to the identical distractors so that the target stimulus would pop out (Treisman and Gelade, 1980; Wolfe and Horowitz, 2004). In the serial search task, however, the target slightly differed from the distractors in either orientation or color. Since the target was not the most salient stimulus in the array and had to be sought using only its remembered appearance, serial attentional search was needed (Buschman and Miller, 2007, 2009). A large body of evidence support that the two types of stimulus configuration in our tasks would induce the participants to make parallel and serial search separately (Treisman and Gelade, 1980; Duncan and Humphreys, 1989; Wolfe et al., 1989; Wolfe and Horowitz, 2004). In fact, the task design analogous to ours was applied by previous primate studies in which monkeys exhibited clearly distinct behavior of parallel and serial search (Buschman and Miller, 2007, 2009).

Each trial began with a 500-ms presentation of a white fixation point (0.2° × 0.2°; luminance 20 Cd/m^2^) in the center of a black background (luminance 0.13 Cd/m^2^). The participants were required to maintain their fixation throughout the trial. The sample of a target to be searched for (length 2°; width 0.2°) was then presented for 1,000 ms, centered on the fixation point, followed by a 500-ms memory delay. Task-irrelevant objects were displayed from the beginning of the delay period until the end of the trial. Following the delay period, an array composed of four search stimuli (length 2°; width 0.2°) positioned 4° vertically or horizontally from the fixation point was presented. The target feature was randomly chosen from nine combinations of three colors (–30°, 0°, or 30° in the hue circle) and three orientations (105°, 135°, or 165° from the vertical meridian) so that each combination was selected at equal frequency through all the trials. In the parallel search task, the distractors were all identical, differing from the target by orientation of 90° and colored as the opposite color of the target color. In the serial search task, the distractors differed independently from the target by either color (30° in the hue circle) or orientation (30°). The difference in color and orientation between the target and the distractors was the same as the difference between targets on different trials. This allowed a target on one trial to be a distractor on the next trial. The position of the target among the four stimulus positions was randomly chosen so that each position was selected at equal frequency through all the trials. The participants then had to report which object was the target by pressing one of four buttons arranged in a square as in the stimulus on a hand-held game pad as quickly as possible. When a button was pressed, or when the elapsed time from the array onset exceeded 1,500 ms without any responses, all the stimuli disappeared, followed by a 1,000-ms interval in which literal feedback was shown in the center of the background.

In each trial, task-irrelevant objects encompassing the four search stimuli were also presented (**Figure 1**). There were two conditions in both tasks: a rectangle condition and cross condition. A previous study found the object-specific advantage even when orthogonally overlapping rectangles were displayed (Shomstein and Yantis, 2002). In the rectangle condition, the objects were composed of three gray-filled rectangles: one long rectangle (12° × 4°; luminance 20 Cd/m^2^) and two small (4° × 4°; luminance 20 Cd/m^2^) rectangles separated by a gap (0.2°), which looked like two long orthogonally overlapping rectangles. In the cross condition, the object was a unitary cross formed by two orthogonally overlapped rectangles (12° × 4°; luminance 20 Cd/m^2^) without a gap, and thus an object-specific advantage was not expected to be observed, since all the line-shaped stimuli were within the same object. The gray rectangles were not mentioned in the instruction to participants, since the object-specific advantage is defined as an attentional effect caused by implicit attention to background objects (Scholl, 2001).

In the rectangle condition, only the rectangle containing the fixation point was expected to receive the object-specific advantage for the following reasons. First, the presentation of a sample stimulus within the rectangle could draw an attentional focus into the location of the stimulus centered on the fixation point. Second, directing spatial attention to a location within the rectangle (“attended rectangle”) could induce the attentional advantage at other locations within the rectangle irrespective of the task requirement to attend to the rectangle itself (e.g., Egly et al., 1994; Moore et al., 1998; Abrams and Law, 2000; Shomstein and Yantis, 2004). Third, the sustained fixation during the delay period could leave spatial attention at the location around the fixation point (i.e., within the rectangle) until search-array presentation. Of course, the 500-ms interval of the delay period might allow participants to shift spatial attention toward the other locations. However, keeping the attentional focus at the fixation point was beneficial for participants to accomplish visual search efficiently, because the target in the search array was randomly presented with equal probability at one of four locations positioned at the equal distance from the fixation point; in addition, participants could know this well through the preceding practice blocks. Thus, we assumed that spatial attention continued to be allocated to the location around the fixation point until search-array presentation and is sufficient to induce the object-specific advantage at all target locations within the attended rectangle containing the fixation point. Note that both accounts of attentional spreading and attentional prioritization are reconciled with this assumption although they make different predictions for the effect of object-specific advantage on stimulus selection in parallel and serial search.

Each participant was seated in a chair placed 60 cm in front of a monitor, and completed six practice blocks of 18 trials and 18 experimental blocks of 18 trials, and each was assigned to either parallel or serial search task.

### PARTICIPANTS

Twenty-four students (seven females and 17 males; 23–29 years old) participated in a single 1-h session. Half were assigned only to the parallel search task, and the other half were assigned only to the serial search task. All participants provided informed consent and the protocol was approved by the Ethics Committee of Nara Institute of Science and Technology. One student assigned to the serial search task was excluded from our analysis because he reported his dyschromatopsia after the experiment. The others all reported normal or corrected-to-normal visual acuity and color vision.

### DATA ANALYSIS

One participant assigned to the serial search task was removed from further analysis because his mean accuracy was 2 SD smaller than the mean accuracy across all the participants. Moreover, we analyzed the RTs, of all the participants for the button press, which were within the correct range of 200–1,500 ms.

We assessed the object-specific attentional advantage in each task by comparing object-based modulations to the target between the cases where the target was presented within an attended rectangle and an unattended one; statistical comparisons were performed using a paired *t*-test (*p* < 0.05) with an effect size computed by the statistical power analysis (Cohen, 1988). The object-based modulation for each task and each participant was calculated as follows. The mean RT across trials (baseline RT) was calculated for each target position in the cross condition where the object-specific advantage was not expected to be observed. Hence, four baseline RTs for individual target positions were obtained. On the other hand, in the rectangle condition, the mean RT across trials was calculated for each target position separately for the cases in which the target appeared within an attended rectangle or within an unattended one. Next, the corrected RTs for individual target positions were calculated by subtracting the baseline RTs from the mean RTs in the rectangle condition so as to eliminate the participant-specific preference for target positions (Buschman and Miller, 2009), and averaged across target positions separately when the target was within the attended rectangle and within the unattended rectangle. Consequently, two averaged values were obtained for each task and each participant. These averaged values are called the object-based modulation.

The effect of object-specific advantage on selection accuracy was calculated in a similar manner. Accuracy, instead of the mean RT, for each target position and each condition was calculated. Corrected accuracies that were obtained by subtracting the baseline accuracies in the cross conditions from the accuracies in the rectangle condition were averaged across target positions separately when the target was within an attended rectangle and within an unattended rectangle. These two averaged values are regarded as the object-based modulation on accuracy.

In addition, we evaluated the object-based modulation using RTs (or accuracies) normalized within each task as percent changes from the baseline RTs (or the baseline accuracies). The percent changes were calculated by dividing the mean RTs (or accuracies) for individual target positions by the baseline RTs (or the baseline accuracies), and averaged over target positions separately for the cases in which the target appeared within an attended or unattended rectangle.

### RESULTS

**FIGURE 2:**
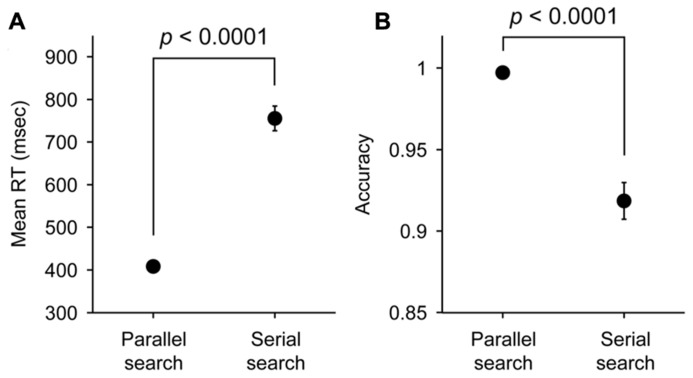
** Mean RTs (A) and accuracy (B) in parallel and serial search tasks**. Error bars represent SEM.

We analyzed the data obtained from 12 participants performing the parallel search task and 10 participants performing the serial search task (**Figure 1**). The mean RT and the accuracy in each task are shown in **Figure 2**. The mean RT acquired in the parallel search task (408 ms) was significantly faster than that acquired in the serial search task (756 ms; two-sample *t*-test, *p* < 0.0001). The accuracy in the parallel search task (0.997) was significantly higher than that in the serial search task (0.919; two-sample *t*-test, *p* < 0.0001). These results indicate that the search efficiency in the serial search task was lower than that in the parallel search task, consistent with previous studies (Treisman and Gelade, 1980; Wolfe and Horowitz, 2004; Buschman and Miller, 2007, 2009).

### OBJECT-SPECIFIC ATTENTIONAL ADVANTAGE IN TWO TASKS

**FIGURE 3:**
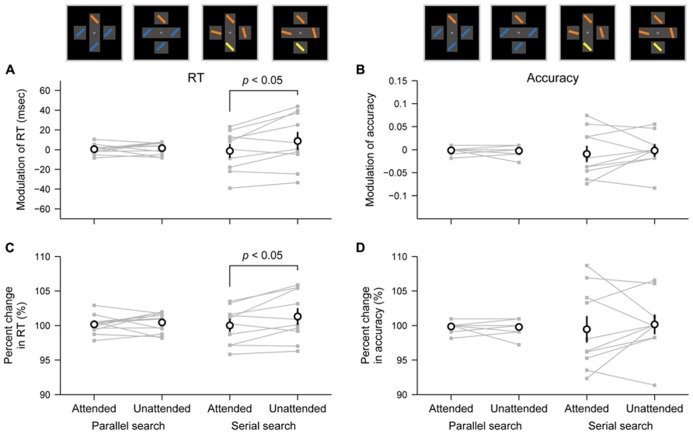
** Differential effects of object-specific attentional advantage between parallel and serial search tasks. (A,B)** Object-based modulation of RT **(A)** and accuracy **(B)** in each pair of tasks (parallel and serial search) and target positions (attended: within an attended rectangle; unattended: within an unattended rectangle). Values reflect behavioral changes that arose from attention to rectangles. **(C,D)** Percent changes in RT **(C)** and accuracy **(D)** relative to the cross condition in each task. Gray-filled plots connected by thin lines represent each participant’s data. Open circles represent averaged values across participants. Error bars represent SEM.

To assess the object-specific attentional advantage in each task, we compared object-based modulations in search performance between the cases in which the target was presented within attended and unattended objects (see Materials and Methods). The object-based modulation of RT in each task is shown in **Figure 3A**. In the serial search task, the modulated RT to the target within an attended rectangle (mean = –1.25 ms) was shorter than the modulated RT to the target within an unattended rectangle (8.79 ms; paired *t*-test, *p* = 0.029, Cohen’s *d* = 0.82). In contrast, in the parallel search task, the difference between the modulated RTs was not significant (attended rectangle, 0.43 ms; unattended rectangle, 1.55 ms; *p* = 0.56, Cohen’s *d* = 0.18). Thus, these results suggest that the object-specific attentional advantage shortens the RT to the target within an attended rectangle relative to the RT to the target within an unattended rectangle only in the serial search task but not in the parallel search task.

The object-based modulation of accuracy in each task is shown in **Figure 3B**. Unlike the results for RT, the difference between modulated accuracies when the target appeared within an attended or unattended object was not significant in both tasks (serial search task, *p* = 0.57, Cohen’s *d* = 0.18; parallel search task, *p* = 0.80, Cohen’s *d* = 0.08). Together, these results suggest that the object-specific attentional advantage facilitates the RT but not the accuracy in the serial search task.

However, one might argue that because the RTs and accuracies were significantly different between two tasks (**Figure 2A**), it would be inappropriate to compare the object-based modulation in the raw RTs or accuracies between the tasks. To address this problem, we also evaluated the object-based modulation using RTs or accuracies normalized within each task as percent changes from the baseline RTs or accuracies (i.e., the mean RTs or the accuracies under the cross condition; see Materials and Methods). Consistent with the results described above, the difference between the modulated RTs was observed only in the serial search task (*p* = 0.035, Cohen’s *d* = 0.78) but not in the parallel search task (*p* = 0.53, Cohen’s *d* = 0.19; **Figure 3C**), and the difference between the modulated accuracies was not significant in both tasks (serial search task, *p* = 0.62, Cohen’s *d* = 0.16; parallel search task, *p* = 0.80, Cohen’s *d* = 0.07; **Figure 3D**).

In addition, our data revealed that the inter-individual variability in object-based modulations changed across the parallel and serial search tasks. The variance of object-based modulations in RTs across participants was larger in the serial search task than in the parallel search task (**Figure 3A**; SD = 23.0 and 4.3 ms, respectively; *F*-test, *p* < 0.0001). Consistent results were observed for the object-based modulations in accuracies (SD = 0.041 and 0.007 for serial and parallel search, respectively), for the percent change in RTs (3.0% and 1.2%), and for the percent change in accuracies (4.58 and 0.68%; **Figures 3B–D**; *p* < 0.005).

### FLOOR EFFECT ON REACTION TIME

We failed to observe the object-specific attentional advantage of RT in the parallel search task in contrast to that in the serial search task (**Figure 3**). However, one might argue that no advantage of RT in the parallel search task was due to a “floor effect”: the baseline RT in the cross condition of the parallel search task (see Materials and Methods) might reach the lower bound of possible response speed for the participants, and the attentional facilitation of the RT when the target appeared within an attended object could not be elicited. If this were the case, the shorter RTs among the RT distribution for each participant would not be more shortened even when the object-specific attentional advantage did yield. However, it would be possible that the longer RTs among the RT distribution were still affected by the object-specific attentional advantage.

**FIGURE 4:**
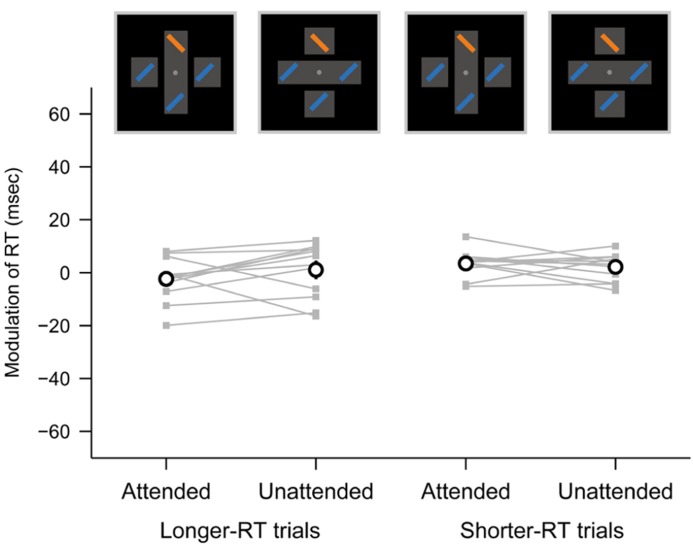
** Object-based modulation of RT in the parallel search task, obtained separately from longer- and shorter-RT trials**. The same conventions are used as in **Figure 3**.

To examine this, we separated the trials in the parallel task into two numerically matched subsets for each target location (top, bottom, left, and right), each object configuration (attended and unattended objects) and each participant according to RT (longer and shorter). The mean RTs across participants were 440 and 374 ms for the longer- and shorter-RT subsets, respectively. We then evaluated the object-based modulation of RT in the parallel search task separately for the RT subsets (**Figure 4**). The object-based modulation did not significantly differ between when the target appeared within attended and unattended objects, not only using the shorter-RT subsets (paired *t*-test, *p* = 0.46) but also using the longer-RT subsets (*p* = 0.21). Thus, the absence of the object-specific attentional modulation in the parallel search task is unlikely to be accounted for by the floor effect.

## DISCUSSION

In this study, to explore the effects of object-based attention and to distinguish attentional spreading and prioritization effects, we developed behavioral tasks by combining the visual search paradigm differentiating parallel and serial search (Buschman and Miller, 2007, 2009) with a variant of the well-established two-rectangle paradigm (Shomstein and Yantis, 2002). Our results revealed that the object-specific advantage was given to the RT in the visual search task inducing serial search but not to that inducing parallel search. This is consistent with the attentional prioritization accounts (Avrahami, 1999; Shomstein and Yantis, 2002, 2004). Therefore, our findings provide evidence to support that the object-specific attentional advantage can be explained by attentional prioritization but not attentional spreading.

The absence of the object-specific attentional advantage on the RTs during the parallel search task could not be due to the much shorter RTs in this task (mean = 408 ms) than those in the serial search task (756 ms; **Figure 2A**). The object-specific advantage on RTs was not observed, even when the RTs were normalized as percent changes (**Figure 3C**) or divided into longer- and shorter-RT groups so as to rule out the possibility of the floor effect (**Figure 4**). Indeed, previous studies have reported the presence of the object-specific advantage when the RTs during their tasks were shorter than the RTs we observed during the parallel search task (e.g., <370 ms in Egly et al., 1994; <390 ms in Avrahami, 1999; <290 ms in Marino and Scholl, 2005). Thus, it seems unlikely that there is too little power to detect the object-specific attentional advantage on RTs during the parallel search task.

Although our result also showed that the object-specific advantage is not given to accuracy of target selection in serial search as well as in parallel search (**Figures 3B,D**), this is consistent with our conclusion. If spatial attention spread within an attended rectangle, an enhanced sensory signal for target stimuli within the rectangle should improve not only RT but also accuracy of target selection. Therefore, the result further supports the absence of attentional spreading within an attended rectangle. In addition, even though the high attentional priority within an attended rectangle affects the order of attentional shifts during serial search, it does not improve sensory sensitivity *per se* and thereby should have no or little effect on accuracy of target selection in contrast to RTs. Hence, the result is also consistent with the prediction from the attentional prioritization account. Nonetheless, there might be a possibility that the change of accuracy by an attentional effect was not evident because of the easiness of the serial search task. However, the RT and accuracy in the serial search task (mean value, 756 ms and 0.919) were sufficiently slower and lower relative to those in the parallel search task (408 ms and 0.997), respectively (**Figure 2**). Thus, the result is unlikely to be an artifact due to task difficulty.

Recently, Lee and Shomstein (2013) provided evidence that space-based and object-based attention is differentially affected by reward. Using the two-rectangle paradigm with reward manipulation, they obtained behavioral and neuroimaging results that space-based attentional allocation was additively modulated by reward bias whereas object-specific attentional advantage was exclusively abandoned by reward bias. Thus, their results imply that object-specific advantage affects a top-down, spatially attentional allocation that is completely replaced with reward-based selection, consistent with our conclusion. In the future, it will be of interest to investigate how the object-specific advantage in serial and parallel search is changed by manipulating reward.

Several studies, however, provided evidence to support the attentional spreading account (Chen and Cave, 2008; Richard et al., 2008). Chen and Cave (2008) applied a variant of the two-rectangle paradigm in which participants were required to report whether two target letters (T or L) at the ends of rectangles were the same or different. In their task, because a spatial cue indicated the possible target locations but always valid, there was likely no need to search for the targets. Their results, however, revealed that the RT was longer when the target letters appeared in an identical object than when they appeared in different objects. Richard et al. (2008) applied a variant of Eriksen’s flanker task (Eriksen and Eriksen, 1974). In their task, the targets and flankers were presented as a circular or rectangular bite which was a part of a connected object or separated multiple objects. Because the locations of the targets and flankers were fixed throughout the experiment, attentional shifts were unlikely to be required. Their results, however, showed that the RT was prolonged when the target and flankers were presented within one connected object compared with when they were presented in separated objects. The findings in these two studies suggest that the objected-specific attentional advantage occurs even when attentional shifts are absent, consistent with the attentional spreading account rather than the attentional prioritization account.

However, the experimental paradigms in these two studies cannot necessarily exclude the effect of attentional shifts during target search for the following two reasons. First, spatial attention is involuntarily captured by stimuli with highly salient properties, despite being irrelevant to the current goals (Yantis and Jonides, 1990). Accordingly, even though the cue and/or certainty of target locations restrict attentional shifts, an attentional focus can be automatically captured by the appearance of target and flanker stimuli, raising the possibility that unexpected attentional shifts may be elicited during search. Second, the processing of target stimuli invoked by their experimental paradigms can be under the control of top-down spatial attention as well as bottom-up spatial attention, because a comparison or discrimination process is required in their paradigms: the comparison of letters (T or L; Chen and Cave, 2008) or the discrimination between circular and rectangular bites (Richard et al., 2008). Hence, the attentional selection of the targets may not be perfectly parallel. In contrast to these paradigms, because the pop-out stimulus in our parallel search task is relevant to the task goal (i.e., target) and, at the same time, can capture spatial attention, no attentional shift is explicitly elicited. Thus, the finding that the object-specific advantage was not found in the parallel search task (**Figures 3 and 4**) suggests that sensory enhancement caused by attentional spreading is unlikely to occur.

Recent studies have reported that object-based representation also enhances the binding of different features within an object even when either of them is task-irrelevant, suggesting that the object-based enhancement of feature binding is automatically operated via attentional spreading across cortical regions that process features within the object (Fiebelkorn et al., 2010; Snyder and Foxe, 2012; Snyder et al., 2012). This indicates that object-based attention interacts with feature-based attention in a bottom-up manner. In contrast, our findings indicate that object-based attention interacts with spatial attention in a top-down manner. One possible reason for this dissociation is that spatial information and object/feature information are processed via distinct visual pathways (dorsal and ventral pathways, respectively) in the cortical system (Ungerleider and Mishkin, 1982; Goodale and Milner, 1992). Feature information may be integrated with object information via a bottom-up process in the ventral pathway of visual stream, whereas spatial information may be integrated with object information via a top-down process in cortical areas in which the dorsal and ventral stream converges, such as the lateral prefrontal cortex (Romanski, 2004; O’Reilly, 2010).

Our results revealed that the inter-individual variability in the object-based modulation of RT and accuracy was larger in the serial search task than in the parallel search task (**Figures 3A,B**) even when the RT and accuracy were normalized within each task as the percent change (**Figures 3C,D**). We speculate that the stronger variability in the serial search task may be caused by the inter-individual difference in the resistance against task-irrelevant information (Kane et al., 2001; Fukuda and Vogel, 2009, 2011; Lechak and Leber, 2011; Kawahara and Kihara, 2011). Because the overlapping rectangles in the rectangle condition of our task had more complex structure than the single cross in the cross condition (**Figure 1**), the participants presumably perceived more visual information in the overlapping rectangles (e.g., multiple segmented rectangles, gaps, depth perception, etc.) even though this information would not distract the object representation of an attended rectangle. Therefore, the participants would be required to suppress this task-irrelevant information to complete visual search efficiently. Such suppression to distracting information involves the lateral prefrontal cortex (Lennert and Martinez-Trujillo, 2011; Suzuki and Gottlieb, 2012), which is also responsible for top-down attentional allocation (Corbetta and Shulman, 2002; Buschman and Miller, 2007; Baluch and Itti, 2011; Squire et al., 2013). Hence, for the participants with weak resistance against distracting information, their cognitive resources in the lateral prefrontal cortex might be allocated more to the suppression of distracting information and less to spatially attentional control, or vice versa, even though the attentional priority assigned to individual stimuli would not be changed. This might lead to the inter-individual variability of the object-based modulation (i.e., the difference of RT or accuracy between the rectangle and cross conditions) in the serial search task. In contrast, in the parallel search task, the bottom-up attentional selection achieved by the function of other cortical areas, such as the posterior parietal cortex (Corbetta and Shulman, 2002; Buschman and Miller, 2007), may be less associated with the suppression of distracting information (Suzuki and Gottlieb, 2012), and show the small variability in the object-based modulation of RT and accuracy across participants.

## AUTHOR CONTRIBUTIONS

Satoshi Nishida and Tomohiro Shibata designed research; Satoshi Nishida performed research and analyzed the data; Satoshi Nishida, Tomohiro Shibata, and Kazushi Ikeda wrote the paper.

## Conflict of Interest Statement

The authors declare that the research was conducted in the absence of any commercial or financial relationships that could be construed as a potential conflict of interest.

## References

[B1] Abrams R. A. Law M. B. (2000). Object-based visual attention with endogenous orienting. *Percept. Psychophys.* 62 818–833 10.3758/BF03206925 10883587

[B2] Avrahami J. (1999). Objects of attention, objects of perception. *Percept. Psychophys.* 61 1604–1612 10.3758/BF03213121 10598473

[B3] Baluch F. Itti L. (2011). Mechanisms of top-down attention. *Trends Neurosci.* 34 210–224 10.1016/j.tins.2011.02.003 21439656

[B4] Buschman T. J. Miller E. K. (2007). Top-down versus bottom-up control of attention in the prefrontal and posterior parietal cortices. *Science* 315 1860–1862 10.1126/science.1138071 17395832

[B5] Buschman T. J. Miller E. K. (2009). Serial, covert shifts of attention during visual search are reflected by the frontal eye fields and correlated with population oscillations. *Neuron* 63 386–396 10.1016/j.neuron.2009.06.020 19679077 PMC2758537

[B6] Chen Z. Cave K. R. (2008). Object-based attention with endogenous cuing and positional certainty. *Percept. Psychophys.* 70 1435–1443 10.3758/PP.70.8.1435 19064488

[B7] Cohen J. (1988). *Statistical Power Analysis for the Behavioral Sciences* 2nd Edn Hillsdale: Lawrence Erlbaum

[B8] Corbetta M. Shulman G. L. (2002). Control of goal-directed and stimulus-driven attention in the brain. *Nat. Rev. Neurosci.* 3 201–215 10.1038/nrn755 11994752

[B9] Duncan J. Humphreys G. W. (1989). Visual search and stimulus similarity. *Psychol. Rev.* 96 433–458 10.1037/0033-295X.96.3.433 2756067

[B10] Egly R. Driver J. Rafal R. D. (1994). Shifting visual attention between objects and locations: evidence from normal and parietal lesion subjects. *J. Exp. Psychol. Gen.* 123 161–177 10.1037/0096-3445.123.2.161 8014611

[B11] Eriksen B. A. Eriksen C. W. (1974). Effects of noise letters upon the identification of a target letter in a nonsearch task. *Percept. Psychophys.* 16 143–149 10.3758/BF03203267

[B12] Fiebelkorn I. C. Foxe J. J. Schwartz T. H. Molholm S. (2010). Staying within the lines: the formation of visuospatial boundaries influences multisensory feature integration. *Eur. J. Neurosci.* 31 1737–1743 10.1111/j.1460-9568.2010.07196.x 20584177

[B13] Fukuda K. Vogel E. K. (2009). Human variation in overriding attentional capture. *J. Neurosci.* 29 8726–8733 10.1523/JNEUROSCI.2145-09.2009 19587279 PMC6664881

[B14] Fukuda K. Vogel E. K. (2011). Individual differences in recovery time from attentional capture. *Psychol. Sci.* 22 361–368 10.1177/0956797611398493 21310945 PMC4494671

[B15] Goodale M. A. Milner A. D. (1992). Separate visual pathways for perception and action. *Trends Neurosci.* 15 20–25 10.1016/0166-2236(92)90344-8 1374953

[B16] Kane M. J. Bleckley M. K. Conway A. R. Engle R. W. (2001). A controlled-attention view of working-memory capacity. *J. Exp. Psychol. Gen.* 130 169–183 10.1037/0096-3445.130.2.169 11409097

[B17] Kastner S. De Weerd P. Desimone R. Ungerleider L. G. (1998). Mechanisms of directed attention in the human extrastriate cortex as revealed by functional MRI. *Science* 282 108–111 10.1126/science.282.5386.108 9756472

[B18] Kawahara J. I. Kihara K. (2011). No commonality between attentional capture and attentional blink. *Q. J. Exp. Psychol.* 64 991–1008 10.1080/17470218.2010.524304 21113860

[B19] Lechak J. R. Leber A. B. (2011). Individual differences in distraction by motion predicted by neural activity in MT/V5. *Front. Hum. Neurosci.* 6: 12 10.3389/fnhum.2012.00012 PMC3279707 22375110

[B20] Lee J. Shomstein S. (2013). The differential effects of reward on space- and object-based attentional allocation. *J. Neurosci.* 33 10625–10633 10.1523/JNEUROSCI.5575-12.2013 23804086 PMC3693052

[B21] Lennert T. Martinez-Trujillo J. (2011). Strength of response suppression to distracter stimuli determines attentional-filtering performance in primate prefrontal neurons. *Neuron* 70 141–152 10.1016/j.neuron.2011.02.041 21482363

[B22] Marino A. C. Scholl B. J. (2005). The role of closure in defining the “objects” of object-based attention. *Percept. Psychophys.* 67 1140–1149 10.3758/BF03193547 16502836

[B23] Moore C. M. Yantis S. Vaughan B. (1998). Object-based visual selection: evidence from perceptual completion. *Psychol. Sci.* 9 104–110 10.1111/1467-9280.00019

[B24] Mozer M. C. Vecera S. P. (2005). “Space- and object-based attention,” in *Neurobiology of Attention* eds Itti L. Rees G. Tsotsos J. K. (New York: Elsevier) 130–134

[B25] Müller N. G. Kleinschmidt A. (2003). Dynamic interaction of object- and space-based attention in retinotopic visual areas. *J. Neurosci.* 23 9812–9816 14586009 10.1523/JNEUROSCI.23-30-09812.2003 PMC6740895

[B26] O’Reilly R. C. (2010). The What and How of prefrontal cortical organization. *Trends Neurosci.* 33 355–361 10.1016/j.tins.2010.05.002 20573407 PMC2916029

[B27] Olson C. R. (2001). Object-based vision and attention in primates. *Curr. Opin. Neurobiol.* 11 171–179 10.1016/S0959-4388(00)00193-8 11301236

[B28] Reynolds J. H. Chelazzi L. Desimone R. (1999). Competitive mechanisms subserve attention in macaque areas V2 and V4. *J. Neurosci.* 19 1736–1753 10024360 10.1523/JNEUROSCI.19-05-01736.1999 PMC6782185

[B29] Richard A. M. Lee H. Vecera S. P. (2008). Attentional spreading in object-based attention. *J. Exp. Psychol. Hum. Percept. Perform.* 34 842–853 10.1037/0096-1523.34.4.842 18665730

[B30] Romanski L. M. (2004). Domain specificity in the primate prefrontal cortex. *Cogn. Affect. Behav. Neurosci.* 4 421–429 10.3758/CABN.4.4.421 15849888

[B31] Scholl B. J. (2001). Objects and attention: the state of the art. *Cognition* 80 1–46 10.1016/S0010-0277(00)00152-9 11245838

[B32] Shomstein S. Behrmann M. (2006). Cortical systems mediating visual attention to both objects and spatial locations. *Proc. Natl. Acad. Sci. U.S.A.* 103 11387–11392 10.1073/pnas.0601813103 16840559 PMC1544095

[B33] Shomstein S. Yantis S. (2002). Object-based attention: sensory modulation or priority setting? *Percept. Psychophys.* 64 41–51 10.3758/BF03194556 11916301

[B34] Shomstein S. Yantis S. (2004). Configural and contextual prioritization in object-based attention. *Psychon. Bull. Rev.* 11 247–253 15260189 10.3758/bf03196566

[B35] Snyder A. C. Fiebelkorn I. C. Foxe J. J. (2012). Pitting binding against selection: electrophysiological measures of feature-based attention are attenuated by Gestalt object grouping. *Eur. J. Neurosci.* 35 960–967 10.1111/j.1460-9568.2012.08016.x 22429245 PMC3413197

[B36] Snyder A. C. Foxe J. J. (2012). The countervailing forces of binding and selection in vision. *Cortex* 48 1035–1042 10.1016/j.cortex.2011.05.003 21665204 PMC3196793

[B37] Squire R. F. Noudoost B. Schafer R. J. Moore T. (2013). Prefrontal contributions to visual selective attention. *Annu. Rev. Neurosci.* 36 451–466 10.1146/annurev-neuro-062111-150439 23841841

[B38] Suzuki M. Gottlieb J. (2012). Distinct neural mechanisms of distractor suppression in the frontal and parietal lobe. *Nat. Neurosci.* 16 98–105 10.1038/nn.3282 23242309 PMC4207121

[B39] Treisman A. M. Gelade G. (1980). A feature-integration theory of attention. *Cogn. Psychol.* 12 97–136 10.1016/0010-0285(80)90005-5 7351125

[B40] Ungerleider L. G. Mishkin M. (1982). “Two cortical visual systems,” in *The Analysis of Visual Behavior* ed. Ingle D. J (Cambridge: MIT Press) 549–586

[B41] Wolfe J. M. Cave K. R. Franzel S. L. (1989). Guided search: an alternative to the feature integration model for visual search. *J. Exp. Psychol. Hum. Percept. Perform.* 15 419–433 10.1037/0096-1523.15.3.419 2527952

[B42] Wolfe J. M. Horowitz T. S. (2004). What attributes guide the deployment of visual attention and how do they do it? *Nat. Rev. Neurosci.* 5 495–501 10.1038/nrn1411 15152199

[B43] Yantis S. Jonides J. (1990). Abrupt visual onsets and selective attention: voluntary versus automatic allocation. *J. Exp. Psychol. Hum. Percept. Perform.* 16 121–134 10.1037/0096-1523.16.1.121 2137514

[B44] Yantis S. Serences J. T. (2003). Cortical mechanisms of space-based and object-based attentional control. *Curr. Opin. Neurobiol.* 13 187–193 10.1016/S0959-4388(03)00033-3 12744972

